# Urine 5-Eicosatetraenoic Acids as Diagnostic Markers for Obstructive Sleep Apnea

**DOI:** 10.3390/antiox10081242

**Published:** 2021-08-03

**Authors:** Hyun-Woo Shin, Kumsun Cho, Chae-Seo Rhee, Il-Hee Hong, Seok Hyun Cho, Sung Wan Kim, Jiyoung Kim, Daeho So, Joo-Youn Cho, Jong-Wan Park

**Affiliations:** 1 Department of Pharmacology, Seoul National University College of Medicine, Seoul 03080, Korea; charlie@snu.ac.kr (H.-W.S.); jikim01@snu.ac.kr (J.K.); specialone@snu.ac.kr (D.S.); 2 Department of Biomedical Sciences, Seoul National University College of Medicine, Seoul 03080, Korea; jasmine615@naver.com (K.C.); joocho@snu.ac.kr (J.-Y.C.); 3 Ischemic/Hypoxic Disease Institute, Seoul National University College of Medicine, Seoul 03080, Korea; 4 Cancer Research Institute, Seoul National University College of Medicine, Seoul 03080, Korea; 5 Department of Otorhinolaryngology-Head and Neck Surgery, Seoul National University Hospital, Seoul National University College of Medicine, Seoul 03080, Korea; csrhee@snu.ac.kr; 6 Metabolomics Medical Research Center (MMRC), Seoul National University College of Medicine, Seoul 03080, Korea; 7 Department of Clinical Pharmacology and Therapeutics, Seoul National University Hospital, Seoul 03080, Korea; 8 Seoul Sleep Clinic, Seoul 06120, Korea; ihh@medimail.co.kr; 9 Department of Otorhinolaryngology-Head and Neck Surgery, Hanyang University College of Medicine, Seoul 04763, Korea; shcho@hanyang.ac.kr; 10 Department of Otorhinolaryngology-Head and Neck Surgery, Kyung Hee University, Seoul 02447, Korea; drkimsw@hanmail.net

**Keywords:** obstructive sleep apnea, hypoxia, urine biomarker, metabolomics, 5-HETE, 5-oxoETE

## Abstract

Early detection of obstructive sleep apnea (OSA) is needed to reduce cardiovascular sequelae and mortality. Full-night polysomnography has been used for diagnosing OSA, but it is too expensive and inconvenient for patients to handle. Metabolome-wide analyses were performed to find and validate surrogate markers for OSA. We further investigated the mechanism underlying hypoxic induction of the markers in human cells and mice. Arachidonic acid derivatives 5-HETE and 5-oxoETE were detected in urine samples. The levels (mean ± SD, ng per mg creatinine) of 5-HETE and 5-oxoETE were 56.4 ± 26.2 and 46.9 ± 18.4 in OSA patients, respectively, which were significantly higher than those in controls (22.5 ± 4.6 and 18.7 ± 3.6). Both levels correlated with the apnea-hypopnea index and the lowest oxygen saturation on polysomnography. After the treatment with the continuous positive airway pressure, the metabolite levels were significantly reduced compared with those before the treatment. In human mononuclear cells subjected to intermittent hypoxia, 5-HETE and 5-oxoETE productions were induced by hypoxia-inducible factor 1 and glutathione peroxidase. When mice were exposed to intermittent hypoxia, 5-HETE and 5-oxoETE were excreted more in urine. They were identified and verified as new OSA markers reflecting hypoxic stress. The OSA markers could be used for OSA diagnosis and therapeutic evaluation.

## 1. Introduction

Obstructive sleep apnea syndrome (OSA), which is characterized by repetitive episodes of upper airway obstructions during sleep, provokes surges in blood pressure and cardiac arrhythmias due to sympathetic activation [1,2,3]. It is also associated with hypercoagulability, vascular oxidative stress, endothelial dysfunction, and systemic inflammation [4]. Thus, OSA patients have life-threatening co-morbidities including strokes and myocardial ischemia. These complications are known to be caused by intermittent hypoxic insult, which is a hallmark of OSA. Therefore, the early detection of OSA is needed to prevent OSA patients from the hypoxia-related complications.

Horrendously, OSA is highly prevalent to affect 17 to 24 percent of the adult population and costs the tremendous economic burden for its diagnosis and treatment [5]. The gold standard for OSA diagnosis is in-laboratory full-night polysomnography. However, as polysomnography is quite expensive and time-consuming, easily applicable tools should be developed to solve the accumulation of patients who require sleep studies [6,7]. Portable monitoring devices have been developed over last few decades, but their benefits in OSA management have been in question. The discovery of diagnostic markers reflecting intermittent hypoxic damages has been a most compelling subject in the OSA research field. Although several candidates from human specimens including serum, urine, saliva, and exhaled breath were suggested [8,9,10], it is unclear whether they are ideal biomarker reflecting hypoxic stress per se independently of accompanying diseases, such as obesity and diabetes [11].

Metabolomic analysis is a powerful method that offers the unbiased identification and state-specific quantification of metabolites. It has developed rapidly into one of the keystones of post-genomic techniques for analyzing molecular signatures, and recently provided a promising solution to uncover the systems of interest at a systems biology level in an unbiased way [12,13,14,15,16]. In this study, we aimed to search and identify OSA markers reflecting the severity of intermittent hypoxic stress.

## 2. Materials and Methods

### 2.1. Pilot, Verification, and Validation Cohorts

We collected the urine samples from male OSA patients at Seoul National University Hospital (SNUH, a tertiary referral center) as a pilot cohort and at Seoul Sleep Clinic (SSC, a local specialized clinic) as a verification cohort (Figures S1 and S2). The control group included healthy volunteers, simple snorers, and insomnia patients. All patients and controls were subjected to full-night in-laboratory polysomnography at the accredited sleep facilities and assisted by certified sleep technicians, and urine samples were collected 7 AM at the end of the polysomnographic recordings and used for metabolomics analysis. In addition, the urine metabolites were rechecked for OSA patients who had been treated with the continuous positive airway pressure (CPAP) in the verification cohort (Table S1, Figure S3). The validation cohort consisted of all patients who were newly diagnosed in four different hospitals (Hanyang University Hospital, Kyoung Hee University Hospital, Seoul National University Bundang Hospital, and Seoul Sleep Clinic) for five months (Figure S4).

### 2.2. Patients and Exclusion Criteria in the Pilot and Verification Cohorts

We collected the urine samples from male OSA patients at Seoul National University Hospital (SNUH, a tertiary referral center) as a pilot cohort and at Seoul Sleep Clinic (SSC, a local specialized clinic) as a verification cohort (Table 1). Newly diagnosed patients between April 2010 and March 2013 were prospectively included in the pilot and verification cohorts. The control group in the pilot and verification cohorts included healthy volunteers, simple snorers, and insomnia patients. All patients and controls underwent full-night in-laboratory polysomnography. Urine samples were collected 7 AM at the end of the nocturnal polysomnographic recordings and were stored at −80 °C for metabolomic analysis. In addition, the urine metabolites were rechecked for OSA patients who were included in the verification cohort and used CPAP during sleep (Table S1). Of eighty OSA patients, 22 were treated with CPAP, 14 with surgery, 11 with oral appliances, 16 with lifestyle modification +/− medication, and 17 refused any treatment. Of 22 patients treated with CPAP, only 11 accepted our proposal for rechecking polysomnography and urine metabolomic analysis as adherents. Adherence to CPAP was defined as CPAP use for 4 h or more daily [17]. According to the CPAP records, the selected patients daily used CPAP for 4.5 h (median) per night (range, 4.0–6.5 h) over the median duration of 34 months (range, 13–37 months). In these cohorts, the subjects were excluded if they had known hypertension, diseases potentially affecting blood pressure control (renal or cardiac transplantation, severe cardiac heart failure), arrhythmia including atrial fibrillation or frequent premature beats (>10/min), smoking, shift work, diabetes mellitus, asthma, chronic obstructive pulmonary disease, history of stroke or myocardial infarction, allergic rhinitis, arthritis, oral appliances or maxillofacial surgery, or pharmacologic treatment that could affect arachidonic derivatives concentration, including aspirin, non-steroidal anti-inflammatory drugs, corticosteroids, and any leukotriene receptor antagonists. This study has not been supported by commercial sponsors. Five of the authors hold a patent application for intellectual property derived from this study.

### 2.3. Patients in the Validation Cohorts

The validation cohort consisted of 18 controls and 102 OSA patients from 4 different hospitals (Hanyang University Hospital [Seoul, Korea], Kyoung Hee University Hospital [Seoul, Korea], Seoul National University Bundang Hospital [Seongnam, Korea] and Seoul Sleep Clinic [Seoul, Korea]) between December 2014 and April 2015 (Table 2). Controls were simple snorers or insomnia patients otherwise healthy. All patients and controls underwent full-night in-laboratory polysomnography. We obtained the urine samples from patients just after PSG. Sixteen patients were under anti-hypertensive medication, and 4 patients were under oral glucose-lowering agents. Four subjects were taking statins against hyperlipidemia.

### 2.4. Cell Culture

Primary human mononuclear cells were purchased from PromoCell (Heidelberg, Germany), and cultured in a specific medium according to the manufacturer’s instruction. Cells were cultured for no more than three passages before the analysis. THP-1, which originated from human peripheral blood monocytes, was obtained from the Korean Cell Line Bank (Seoul, Korea). THP-1 cells were cultured in RPMI-1640 supplemented with 10% heat-inactivated fetal bovine serum. Cells were grown in 5% CO_2_/21% O_2_ (normoxic) or in 5% CO_2_/1% O_2_ (hypoxic) conditions, and the conditioned media were collected for metabolomic analysis. In addition, siRNAs and chemical inhibitors of hypoxia-inducible factor 1 (HIF-1) was used to evaluate the role of HIF-1 in generation of target metabolites.

### 2.5. Animals and Hypoxic Exposures

The Institutional Animal Use and Care Committee of Seoul National University approved this animal study (SNU-140103-1). Male C57BL/6J mice (18–20 g) were purchased from Central Laboratory Animal Inc. (Seoul, Korea). According to the experimental protocols provided by the Institutional Animal Use and Care Committee, all efforts were made to minimize animal suffering and to reduce the number of animals used. To induce an intermittent hypoxia, mice were maintained in hypoxic chambers (Oxycycler model A44XO, BioSpherix, Redfield, NY, USA) operated under a 12 h light-dark cycle (7:00 a.m.–7:00 p.m.) for 14 days with periodical urine collection, as previously reported [18,19]. Mice were randomly assigned to three different conditions of intermittent hypoxia (IH), continuous hypoxia (CH), or room air (RA) exposures. Hypoxic exposures were kept every day from 9 a.m. to 5 p.m. for 8 h. Urine samples were collected just after the end of hypoxic stimulus and were stored at −80 °C.

### 2.6. Metabolite Analyses Using LC/Q-TOF MS

The defrosted urine samples (100 μL) were diluted to four volumes of cold water in 1.5 mL e-tubes. After brief vortex mixing, supernatants were collected after centrifugation at 14,000× *g* for 20 min at 4 °C, and transferred to vials for LC/Q-TOF MS analysis. Chromatographic separations of metabolites in urine were performed with a Zorbax SB-C18, 50 mm × 2.1 mm, 1.8 μm (Agilent Technologies, Santa Clara, CA, USA) analytical column using an Agilent 1200 series (Agilent Technologies, Santa Clara, CA, USA). A 5 μL of diluted urine sample was loaded onto the column held at 40 °C and eluted with 0.1% formic acid and 2 mM ammonium formate in water (Solvent A), and 0.1% formic acid in methanol (Solvent B) over 21 min. While maintaining a constant flow rate of 0.4 mL/min, the metbolites were eluted using the following gradients of 2–98% B from 0.1 to 13 min, and 98% B was held constant for 2 min followed by a return to 2% B from 15.1 to 17 min. The eluent was introduced into the mass spectrometer (Agilent 6530 quadrupole time-of-flight (Q-TOF) mass spectrometer, Agilent Technologies, Santa Clara, CA, USA) by electrospray ionization (ESI), with ESI Vcap, MS TOF fragment, MS TOF skimmer, and nozzle voltages set in the positive ion mode to 3500, 170, 65, and 1000 V, respectively. The nebulizer gas and drying gas was set to 30 psig and 11 μL/min, respectively, and ESI gas temperature was 325 °C. Centroid data were acquired over an *m*/*z* range of 100–1100 using an accumulation time of 0.25 s per spectrum. All spectra were mass corrected in real time by external reference through an independent reference electrospray. For each injection batch, the overall quality of the analysis procedure was monitored using repeat extracts of a pooled urine sample.

### 2.7. Quantification of Biomarkers

MassHunter Quantitative analysis (Agilent Technologies) was used to quantify 5-HETE and 5-oxo-ETE, of which authentic compounds were commercially available. Creatinine was also quantified to normalize to the actual concentrations of each urinary biomarker. One hundred microliters of urine supernatant was diluted with 900 μL of three internal standard mixtures: 2.5 ng/mL of 5-HETE-d_8_ ([M + H]^+^ = 329.2926) for 5-HETE, and of 5-oxo-ETE-d_7_ ([M + H]^+^ = 326.2707) for 5-oxo-ETE, and 50 μg/mL of 1,3-dimethyl-2-imidazolidinone ([M + H]^+^ = 115.0866) for creatinine. Calibration curves were constructed from 0.5 to 10 ng/mL of 5-HETE-d_8_ and 5-oxo-ETE-d_7_, and from 10 to 1000 μg/mL of 1,3-dimethyl-2-imidazolidinone. The concentration of each biomarker in urine was determined from the calibration curves using linear regression analysis. All determined correlation coefficients were >0.99 for each biomarker, and the resultant concentrations were expressed as ng/mg creatinine (normalized).

### 2.8. Immunoblotting

To quantify protein levels, total proteins were separated on SDS/polyacrylamide gels, and transferred to Immobilon-P membranes (Millipore, Billerica, MA, USA). Membranes were then blocked with 5% nonfat milk in Tris-buffered saline containing 0.05% Tween-20 (TTBS) at room temperature for 1 h, and incubated overnight at 4 °C with a primary antibody diluted 1:1000 to 1:5000 in 5% nonfat milk in TTBS. Horseradish peroxidase-conjugated anti-rabbit antiserum was used as a secondary antibody (1:5000), and antigen-antibody complexes were visualized using an ECL Select kit (GE healthcare, Pittsburgh, PA, USA). Rabbit polyclonal anti–HIF-1α antiserum was generated in our laboratory [20], and anti-tubulin serum was purchased from Santa Cruz Biotechnology (Santa Cruz, CA, USA).

### 2.9. Glutathione Peroxidase Activity

Commercially available enzyme assay kits were used to determine the activity of glutathione peroxidase GPX activity (ab102530, Abcam, Cambridge, MA, USA). Cells were grown in the presence of indicated normoxic or hypoxic conditions for 24 h, and lysates prepared from 2 × 10^6^ cells according to the manufacturer’s instructions. Enzyme activity was determined using a Power Wave HT microplate spectrophotometer (BioTek Instruments, Seoul, Korea), and the results were normalized to the total protein concentration.

### 2.10. siRNA and Plasmid DNA Transfection

Cells at 40% confluence were transfected with siRNAs using RNAiMAX reagent (Invitrogen, Waltham, MA, USA) according to the manufacturer’s instructions. The sequences of siRNAs (IDT, Iowa) were 5′-CAAAGUUAAAGCAUCAGG-3′ and 5′-GAAGGAACCUGAUGCUUU-3′ for HIF-1α. Stealth RNAi negative control duplex (Invitrogen) was used as a control siRNA.

### 2.11. Statistics

Comparisons between patients with OSA and control subjects for continuous variables were made by the Mann–Whitney U test. To compare outcomes among three groups, the ANOVA was used. The mass abundance of metabolites from in vitro, in vivo and human specimens was also compared using the Mann–Whitney U test. The Wilcoxon signed rank test was used to evaluate the change of candidate markers in samples between baseline and post-CPAP values. Receiver-operating-characteristic (ROC) analysis was used to quantify the diagnostic values of the candidate biomarker. In addition, we conducted multivariate logistic regression analyses to estimate the odds ratio (OR) of OSA (AHI ≥ 5) in relation to cutoff values of 5-HETE and 5-oxoETE with a 95% confidence interval (CI). The potential confounding variables included in the multivariate model were age, gender, body mass index, smoking status, and presence of hypertension and diabetes mellitus. All statistical analyses were performed by using IBM SPSS Statistics version 21.0 (Chicago, IL, USA). *p* < 0.05 was considered as significant.

## 3. Results

### 3.1. Characteristics of the Study Population

During a three-year period (April 2010 to March 2013), male patients (96 in the pilot cohort; 90 in the verification cohort) underwent full-night in-laboratory polysomnography and participated in the study. Urine samples form 143 participants of them were adequately collected and subjected to metabolomics analysis. During a five-month period (December 2014 to April 2015), 169 male and female patients were enrolled in the validation cohort study, and finally polysomnography and urine metabolomics data of 120 participants were analyzed.

### 3.2. Identification of Urine 5-HETE as an OSA Marker

**Figure 1:**
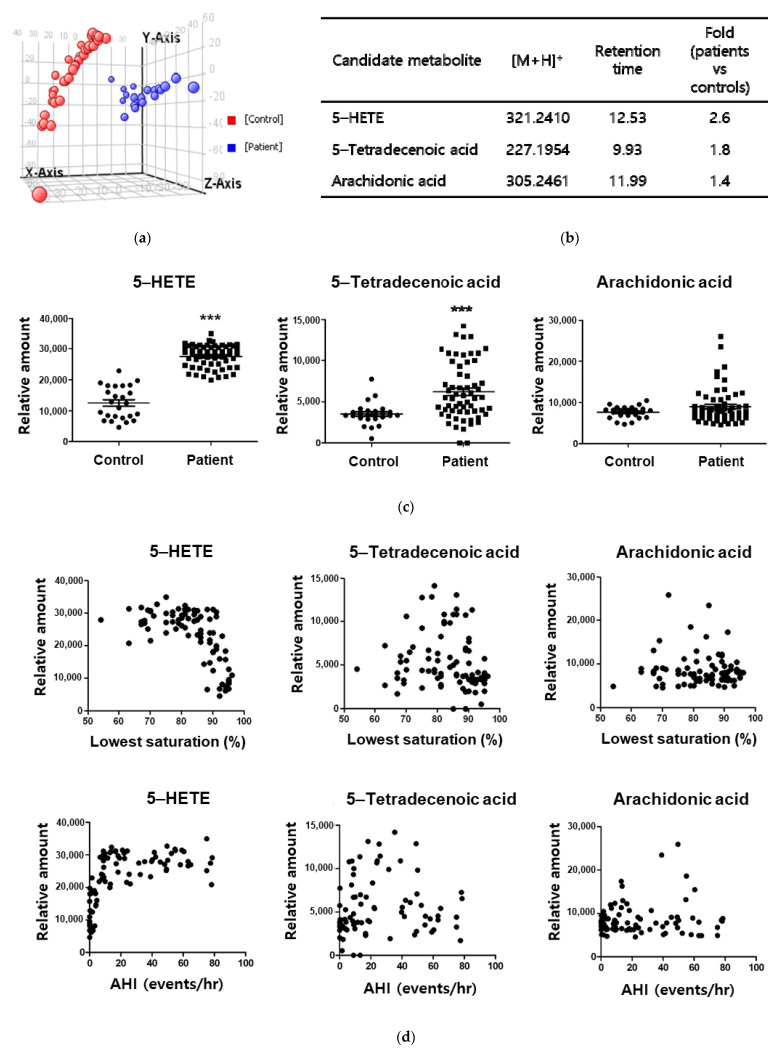
Identification of 5-HETE as a hypoxic biomarker in the obstructive sleep apnea syndrome. (**a**) Results of the partial least squares discriminant analysis of urine specimens from patients with the obstructive sleep apnea syndrome and controls. In a pilot cohort, moderate to severe OSA patients (*n* = 20) with AHI > 10 were compared to the simple snorers or healthy subjects as controls (*n* = 38) with AHI < 5. The peaks of 5-HETE, which was one of the crucial urine metabolites distinguishing two groups, were visualized in comparison with the standard material of it. (**b**) List of three major metabolite biomarkers for the obstructive sleep apnea, which were identified and significantly higher in urine specimens from OSA patients than in those from controls. (**c**) Levels of three identified metabolites in a different group as a verification cohort—25 healthy controls, 60 OSA patients. The horizontal lines indicate means, and I bars are standard deviations. (**d**) Correlation between the levels of candidate metabolite markers and the lowest oxygen saturation or apnea-hypopnea index (AHI) from polysomnographic studies in the verification cohort.

We collected the first morning urines that contain metabolites accumulated during sleep, and compared urine metabolite profiles between 20 patients with moderate to severe OSA and 38 controls in the pilot cohort (Table 1). A partial least squares-discrimination analysis (PLS-DA) reveals that urinary metabolic profiles are significantly different between OSA and control groups (Figure 1a). Based on the profiles (Figure 1b), 5-tetradecenoic acid, arachidonic acid and 5-hydroxyeicosatetraenoic acid (5-HETE) were considered as potential OSA markers. Confirmatory assessments showed that 5-tetradecenoic acid and 5-HETE increase markedly in OSA patients (a verification cohort in Table 1; Figure 1c). Of two metabolites, 5-HETE has a better correlation with lowest oxygen saturation and apnea-hypopnea index (AHI) recorded in polysomnography (Figure 1d) rather than either body mass index (BMI) or age (Figure S5a). Considering the known correlation between OSA and BMI or age, we performed the partial correlation test, which revealed that 5-HETE level correlates with lowest oxygen saturation and AHI even after controlling BMI and/or age (Figure S5b).

### 3.3. Analyses of Metabolites in the 5-Lipoxygenase Pathway

**Figure 2:**
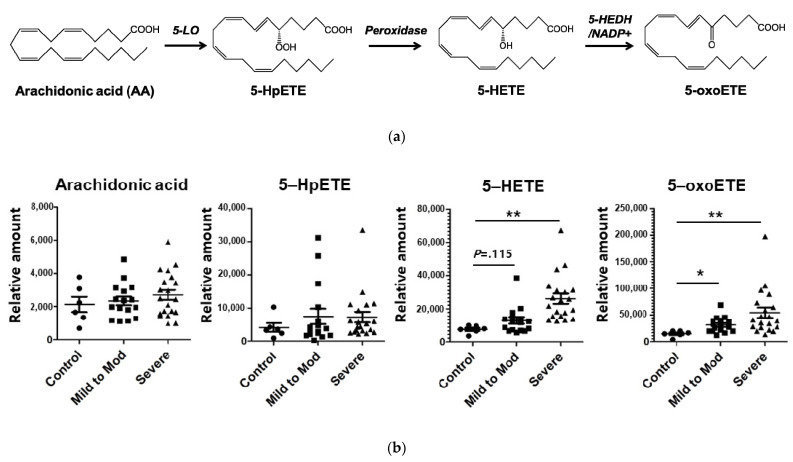

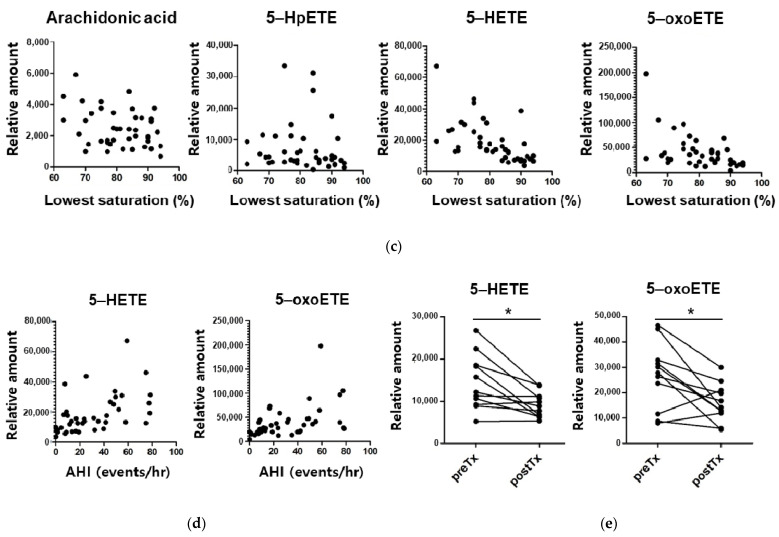
Levels of arachidonic acid derivatives in 5-lipoxygenase pathway and the association with the oxygen desaturation. (**a**) Arachidonic acid pathway and its derivatives through 5-lipoxygenae, glutathione peroxidase, and 5-hydroxyeicosanoid dehydrogenase. (**b**) Shown are the levels of arachidonic acid and its derivatives according to the severity of the obstructive sleep apnea syndrome—healthy control (apnea-hypopnea index [AHI] < 5), mild to moderate patients (5 ≤ AHI < 30), and severe patients (30 ≤ AHI). The horizontal lines indicate means, and I bars are standard deviations. (**c**,**d**) Correlation of arachidonic acid and its derivatives with the lowest oxygen saturation or with AHI. (**e**) Change of 5-HETE and 5-oxoETE in urine from the patients with the obstructive sleep apnea after the continuous positive airway pressure (CPAP) treatment. * and ** mean *p* < 0.05 and *p* < 0.01, respectively.

Given the metabolic pathway of 5-HETE (summarized in Figure 2a), we performed the targeted analysis to quantify arachidonic acid, 5-hydroperoxyeicosatetraenoic acid (5-HpETE), 5-HETE, and 5-oxo-6E,8Z,11Z,14Z-eicosatetraenoic acid (5-oxoETE). Further, 5-HETE and 5-oxoETE increased in the mild-to-moderate OSA group and more in the severe OSA group patients (Figure 2b). Arachidonic acid and 5-HpETE marginally increased in the OSA groups but the differences were not significant. Although each of four metabolites showed a tendency of negative correlation with the lowest oxygen saturation, 5-HETE and 5-oxoETE had higher correlation coefficients than arachidonic acid and 5-HpETE (Figure 2c). The 5-HETE and 5-oxoETE also correlated with the apnea-hypopnea index (Figure 2d), but arachidonic acid and 5-HpETE did not (Figure S6). Next, we investigated whether 5-HETE and 5-oxoETE levels were reduced after appropriate CPAP treatment. The effect of CPAP on oxygenation in OSA patients was verified by checking oxygen profiles on polysomnography (Table S1). After patients used CPAP for more than a year, 5-HETE and 5-oxoETE levels were significantly reduced compared with those before CPAP treatment (Figure 2e). Patients’ body mass indexes were little changed after CPAP treatment (Table S1).

### 3.4. Verification and Validation of 5-HETE and 5-oxoETE as Diagnostic Markers for OSA

**Figure 3:**
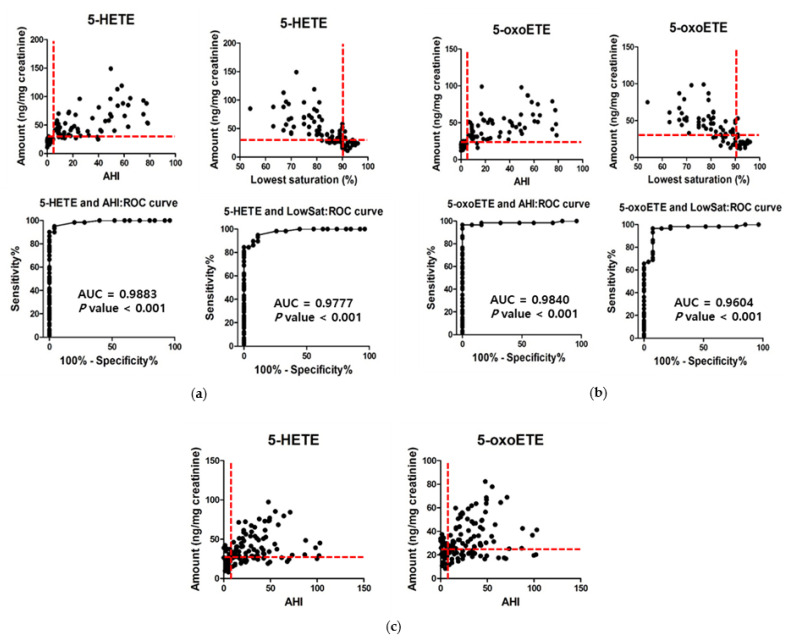
Verification of 5-HETE or 5-oxoETE as OSA markers. (**a**,**b**) Amount of 5-HETE or 5-oxoETE in urine from a verification cohort and their correlations with AHI or the lowest saturation on polysomnography. Receiver-operating-characteristic (ROC) analysis was performed to quantify the diagnostic values of each candidate biomarker. AUC, the area under curve. (**c**) Amount of 5-HETE or 5-oxoETE in urine from a validation cohort and their correlations with AHI or the lowest saturation on polysomnography.

To assess the most effective cutoff value for 5-HETE and 5-oxoETE, we performed the absolute quantification using their standard compounds and computed a receiver-operating-characteristic (ROC) curve. For 5-HETE, the area (±SE) under the ROC curve was 0.988 ± 0.008 (95% confidence interval [CI], 0.972 to 1.004; *p* < 0.001) with the diagnostic criteria of AHI > 5. At the cutoff value of 27.5 ng 5-HETE/mg creatinine, the sensitivity was 95%, and the specificity was 96% (Figure 3a). For 5-oxoETE, the area (±SE) under the ROC curve was 0.984 ± 0.014 (95% confidence interval [CI], 0.957 to 1.011; *p* < 0.001) with the diagnostic criteria of AHI > 5. At the cutoff value of 24.0 ng 5-oxoETE/mg creatinine, the sensitivity was 97%, and the specificity was 92% (Figure 3b). In addition, with the diagnostic criteria of the lowest oxygen saturation <90%, the areas under the ROC curves for 5-HETE and 5-oxoETE were 0.978 ± 0.012 (95% confidence interval [CI], 0.954 to 1.002; *p* < 0.001) and 0.960 ± 0.021 (95% confidence interval [CI], 0.918 to 1.003; *p* < 0.001), respectively. This indicates urinary 5-HETE and 5-oxoETE could be reliable diagnostic markers for OSA.

Following this, we investigated the diagnostic values for these cut off values of urinary 5-HETE and 5-oxoETE in a validation cohort including OSA patients with hypertension, diabetes, and other OSA-associated diseases (Table 2). As expected, 5-HETE and 5-oxoETE levels in urine samples correlate with the apnea-hypopnea index in this cohort (Figure 3c). At the cutoff value of 27.5 ng 5-HETE/mg creatinine, the sensitivity was 75% and the specificity was 72%. At the cutoff value of 24.0 ng 5-oxoETE/mg creatinine, the sensitivity and specificity were 67% and 61%, respectively. When we adopted the cutoff values of AHI for 5-HETE and 5-oxoETE as above, both sensitivity and specificity were decreased, but still applicable to screening objectives. To estimate the odds for OSA (AHI ≥ 5) in relation to cutoff values of 5-HETE and 5-oxoETE, a logistic regression analysis was conducted (Table 3). In the unadjusted analysis, subjects who had 5-HETE (≥27.5 ng/mg creatinine) and 5-oxoETE (≥24.0 ng/mg creatinine) had 7.60-fold (95% CI, 2.47–23.37) and 3.29-fold (95% CI, 1.17–9.25) increased odds for OSA, compared to those who had 5-HETE (<27.5 ng/mg creatinine) and 5-oxoETE (<24.0 ng/mg creatinine), respectively. In multivariate analysis, subjects whose 5-HETE and 5-oxoETE levels exceed the corresponding cutoff values showed a further increased OR of OSA.

### 3.5. Mechanism Study in Human Mononuclear Cells

**Figure 4:**
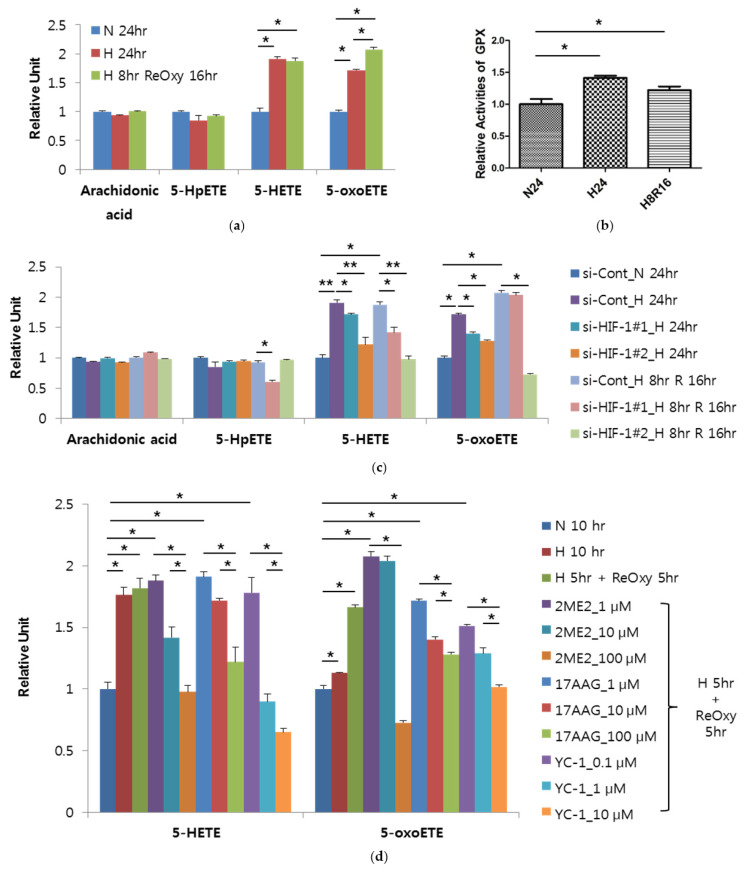
Augmented secretion of 5-HETE and 5-oxoETE from human monocytes by hypoxia. (**a**) Levels of arachidonic acid and its derivatives secreted from THP-1, a human monocyte cell line, by normoxia (N, 21% O_2_ for 24 h), hypoxia (H, 1% O_2_ for 24 h) or reoxygenation (ReOxy, 21% O_2_ for 16 h) after hypoxia (H, 1% O_2_ for 8 h). (**b**) Activity of glutathione peroxidase in primary human mononuclear cells under normoxic, hypoxic, or reoxygenation after hypoxia conditions. (**c**) Silencing of hypoxia-inducible factor 1α (HIF-1α) reduced the secretion of 5-HETE and 5-oxoETE from THP-1 cells by normoxia (N, 21% O_2_ for 24 h), hypoxia (H, 1% O_2_ for 24 h) or reoxygenation (R, 21% O_2_ for 16 h) after hypoxia (H, 1% O_2_ for 8 h). (**d**) HIF-1 inhibitors including 2-methoxyestradiol (2ME2), 17-(Allylamino)-17-demethoxygeldanamycin (17-AAG) and YC-1 inhibited 5-HETE and 5-oxoETE secretion from THP-1 cells. THP-1 cells were incubated in normoxia (N, 21% O_2_ for 10 h), hypoxia (H, 1% O_2_ for 10 h) or reoxygenation (R, 21% O_2_ for 5 h) after hypoxia (H, 1% O_2_ for 5 h). * *p* values < 0.05; ** *p* values < 0.01.

Myeloid lineage cells are the main sources that produce pro-inflammatory cytokines synthesized through the arachidonate 5-lipoxygenase pathway [21]. Indeed, a previous report demonstrated that rat alveolar macrophages secreted 5-HETE under hypoxia [22], which encouraged us to test the possibility that 5-HETE or 5-oxoETE secretion from human mononuclear cells could be stimulated by hypoxia. In addition, sleep apnea is a condition in which repeated reperfusion injury can occur because it is accompanied by severe hypoxia and rapid reoxygenation of blood intermittently. Therefore, in order to satisfy similar physiological conditions, the THP-1 human monocyte cell line was incubated under 8 h-hypoxia followed by 16 h-reoxygenation, in addition to chronic hypoxia environment by 24 h-hypoxia (1% O_2_). Compared to the normoxia group, both metabolites were more secreted in either hypoxia or hypoxia-reoxygenation group (Figure 4a). Next, we rechecked the hypoxic secretion of the metabolites in human peripheral blood mononuclear cells (hPBMCs). Considering sleeping duration, we set the incubation time for intermittent or continuous hypoxia to 8 h. For intermittent hypoxia, cells were exposed to 8 cycles of hypoxia and normoxia, and each cycle consisted of 10 min-hypoxia followed by 50 min-reoxygenation. As a result, 5-HETE and 5-oxoETE levels in culture media were increased after either intermittent or continuous hypoxia, whereas arachidonic acid and 5-HpETE were not altered (Figure S7a). Moreover, the activity of glutathione peroxidase, which converts 5-HpETE to 5-HETE, was increased in hPBMCs exposed to hypoxia or hypoxia-reoxygenation (Figure 4b), suggesting that the hypoxia-induced production of 5-HETE and 5-oxoETE may attribute to the activation of glutathione peroxidase. Hypoxia-inducible factor 1 (HIF-1), which transcribes 100 or more genes essential for hypoxic responses, has been reported to induce the inflammatory mediator production in mononuclear cells [22,23]. Hence, we investigated whether the hypoxic secretion of 5-HETE and 5-oxoETE secretion depends on HIF-1α. Two different siRNAs, which effectively downregulated HIF-1α under hypoxia (Figure S7b), attenuated the hypoxic secretion of 5-HETE and 5-oxoETE in THP-1 cells and hPBMCs (Figure 4c and Figure S8C). In addition, HIF-1 inhibitors 2-methoxyestradiol (2ME2), 17-(Allylamino)-17-demethoxygeldanamycin (17-AAG) and YC-1 significantly blocked 5-HETE and 5-oxoETE secretion in THP-1 cells exposed hypoxia-reoxygenation (Figure 4d). These results support the involvement of HIF-1α in the hypoxia-induced production of 5-HETE and 5-oxoETE.

### 3.6. Verification of 5-HETE and 5-oxoETE as Intermittent Hypoxia Markers in Mice

**Figure 5:**
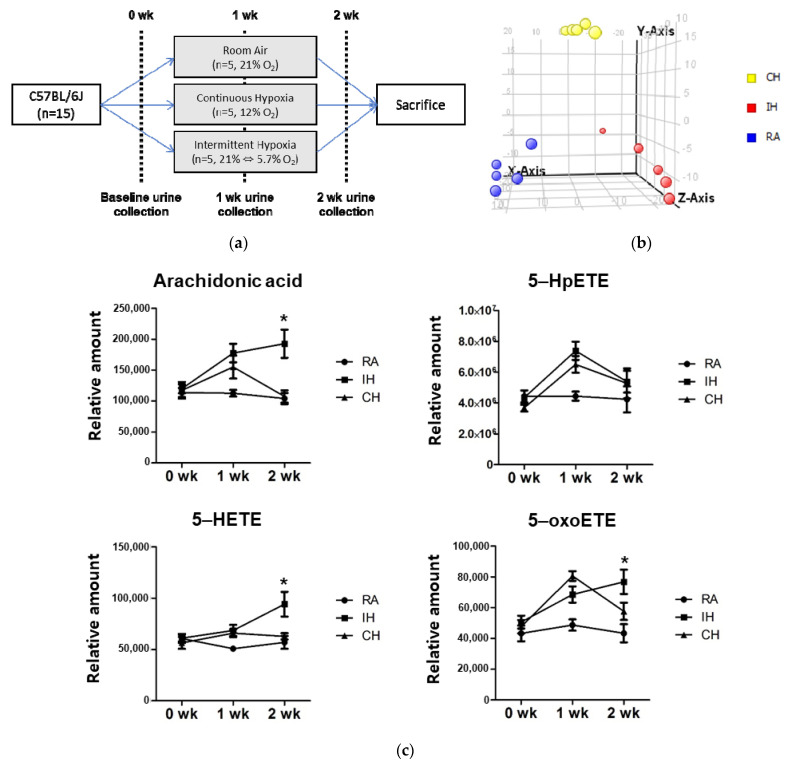
Changes of urine metabolites induced by hypoxic stimulus in a murine model. (**a**) The schema of the urine collection form the mice after exposure of room air (RA), continuous hypoxia (CH, 12% O_2_), or intermittent hypoxia (IH, alternating period of 21% and 5.7% O_2_) for 2 wks). (**b**) Results of the partial least squares discriminant analysis of urine specimens from the mice, which were exposed to three different oxygen surroundings. (**c**) Change of arachidonic acid and its derivatives 5-HpETE, 5-HETE and 5-oxoETE in mice urine at baseline, 1 and 2 wks after the treatment. * *p* values < 0.05.

As shown in Figure 5a, 15 mice (5 per group) were subjected to room air (RA), continuous hypoxia (CH, 12% O_2_), or intermittent hypoxia (IH, alternating period of 21% and 5.7% O_2_) for two weeks. Urine samples were collected before hypoxic exposure (baseline), and after one or two week-exposure. PLS-DA analysis revealed that those three groups have distinct urinary metabolic profiles (Figure 5b). Arachidonic acid, 5-HETE and 5-oxoETE increased after two week-exposure to intermittent hypoxia, but 5-HpETE did not significantly (Figure 5c). The levels of urine 5-HpETE and 5-oxoETE are likely to reflect two week-exposure to intermittent hypoxia in mice. These results further verify 5-HpETE and 5-oxoETE as surrogate markers for OSA diagnosis.

## 4. Discussion

In this study, we found that 5-HETE and 5-oxoETE increase significantly in urine samples from OSA patients and that their levels correlate with hypoxia indexes on polysomnography. These metabolites are secreted from human mononuclear cells in response to hypoxia or hypoxia-reoxygenation, and also increased in urines of mice exposed to intermittent hypoxia for two weeks. Based on these results, we propose that urine 5-HETE and 5-oxoETE be used as hypoxia markers for OSA diagnosis.

Intermittent hypoxia is a hallmark feature of OSA and evokes sympathetic activation and systemic inflammation [24,25]. Although the pathogenesis of cardiovascular complications by OSA is not fully understood, endothelial dysfunction due to systemic inflammation is believed as an etiology underlying the complications [26]. We here screened for OSA markers through metabolome-wide random approach and identified 5-HETE and 5-oxoETE as the candidates. These metabolites are synthesized via the arachidonate 5-lipoxygenase pathway, which is well known to be activated under inflammation. Given that OSA provokes systemic inflammation, it is not surprising that the arachidonate-derived metabolites accumulate in OSA patients. Indeed, a few recent studies have demonstrated that 5-HETE and 5-oxoETE are secreted from human leukocytes during oxidative stress [27,28]. To our best knowledge, however, the metabolites have not been suggested as OSA markers.

The 5-HETE synthesis from 5-HpETE is catalyzed by glutathione peroxidase (GPX), and then 5-HETE are converted to 5-oxoETE by 5-hydroxyeicosanoid dehydrogenase (5-HEDH). In mononuclear cells, continuous or intermittent hypoxia promoted GPX activation, which may underlie increased production of 5-HETE and 5-oxoETE. Although GPX activity has not been checked in OSA patients, a previous report showed that plasma GPX activity and GSH level are significantly higher in breath-hold divers who has been chronically exposed to intermittent hypoxia [29]. Moreover, HIF-1, which orchestrates cellular responses to hypoxia, was found to be responsible for 5-HETE and 5-oxoETE induction under continuous or intermittent hypoxia. Given a previous report showing that the plasma GPX expression is directly regulated by HIF-1 through its promoter region [30], it is speculated that the in mononuclear cells exposed to hypoxia HIF-1 stimulates 5-HETE and 5-oxoETE productions by upregulating GPX. This metabolic pathway remains to be further investigated.

The 5-lipoxygenase pathway is the main process to convert arachidonate to 5-HpETE and leukotrienes (LTs). LTs are potent proinflammatory mediators that play crucial roles in inflammatory diseases [31]. The LT family includes LTB4 and cysteinyl leukotrienes (cysLTs: LTC4, LTD4, and LTE4), which display specific and overlapping functions as inflammatory mediators [32]. In fact, LTB4 has been reported to increase in OSA patients and to be linked with early vascular remodeling and atherosclerosis [33]. We also checked the LTB4 level in urines and found that the mean value of LTB4 is higher in OSA patients than in controls (Figure S8a). However, the LTB4 levels were not only widely distributed among OSA patients but also irrelevant to the lowest oxygen saturation (Figure S8b). In addition, it has been reported that urinary LTE4 level correlates with obesity and metabolic disorder rather than with sleep hypoxia [8]. Of arachidonate metabolites, 8-isoprostane has been also reported to increase in OSA patients [34,35,36]. As isoprostane synthesis is triggered under oxidative conditions, isoprostanes have been used to evaluate oxidative stress in vivo [37]. Given that reactive oxygen species are generated at the reoxygenation phase during intermittent hypoxia, it is reasonable that the oxidative stress-induced metabolite increases in OSA patients. However, the large population study showing its correlation with hypoxic parameters remains to be designed for the application of 8-isoprostane to OSA diagnosis. In addition, the 8-isoprostane levels in serum, urine or exhaled condensates were as variable as they were overlapped between control and patient groups [34,35]. Compared to the metabolites suggested previously, 5-HETE and 5-oxoETE have a better correlation with hypoxia parameters, and thus, could be specific markers for OSA diagnosis. Moreover, as their levels are not so much overlapped between control and patient groups, they can be used for diagnosing OSA with higher sensitivity and specificity. Finally, the logistic regression analysis revealed the quite high odds ratio in the general OSA population (validation) cohort.

There are few reports on the role of 5-HETE and 5-oxoETE in terms the cardiovascular consequences. Further, 5-HETE is known as chemotactic for human neutrophils [38], and inhibited PGI2 production in porcine coronary artery endothelial cells [39]. Both 5- and 15-HETE induced pulmonary edema, possibly due to increased lung vascular permeability [40]. High concentrations of HETE, including 5-HETE, 12-HETE, and 15-HETE, were reported in atherosclerotic plaques, especially in those that were more likely to rupture [41]. Elevated concentrations of circulating 5-HETE and 12-HETE were observed in patients after cardiac surgery [42]. Elevated concentrations of 5-HETE, 12-HETE, and 15-HETE were reported in individuals with acute cardiac syndrome [43]. Elevated circulating concentrations of 5-HETE, 12-HETE, and 15-HETE were reported in patients with coronary arterial disease [44]. Considering its effects on neutrophils and monocytes, 5-oxo-ETE could also be involved in cardiovascular disease [45].

This study has a few drawbacks in methodological aspects. To validate the clinical efficacy of these markers thoroughly, we firstly need to increase the cohort size, or evaluate them in different ethnic groups or in a wide range of BMI groups. Secondly, this study showed current levels of metabolites correlating with the polysomnographic parameters, but provided no evidence supporting the potential linkage of the metabolites with OSA complication or mortality. This should be investigated in the future prospective research by long-term follow-up. Finally, the development of convenient tools for detecting these urinary metabolites has not been established, so the quantification depends on highly sophisticated equipment like LC-MS. Although the ELISA kit for serum 5-HETE, not for 5-oxoETE, is commercially available, it was not applicable to urine 5-HETE in our experimental setting (data not shown). The development of more convenient methodology is mandatory for the widespread utilization of these novel markers for OSA diagnosis. With the recent development of targeted metabolomics technology, the cost of analysis can be significantly reduced when performing mass analysis. In addition, if a diagnostic kit that detects a target metabolite is made, it can be used more widely.

## 5. Conclusions

Our observations suggest that urinary 5-HETE and 5-oxoETE productions are induced from human mononuclear cells exposed to hypoxia, and their urinary levels positively correlate with hypoxic severity in OSA patients. Moreover, the metabolite levels in OSA patients are reduced after appropriate CPAP treatment. Based on these results, we here propose that 5-HETE and 5-oxoETE can be used as surrogate makers to diagnose OSA and to evaluate the disease progress or the treatment effectiveness.

## Figures and Tables

**Table 1:** Baseline characteristics of patients with the obstructive sleep apnea syndrome and controls *.

	Pilot Cohort			Verification Cohort		
Characteristic	Control	Patients with OSA	*p* Value	Control	Patients with OSA	*p* Value
*n*	38	20	-	25	60	-
Age (yrs)	32.0 ± 10.9	39.4 ± 13.5	0.040	36.4 ± 10.3	38.8 ± 7.4	0.067
BMI (kg/m^2^) †	21.9 ± 3.1	25.1 ± 3.0	<0.001	24.8 ± 1.9	25.9 ± 2.8	0.068
Neck circumference (cm)	34.7 ± 3.2	38.0 ± 3.1	<0.001	38.4 ± 1.9	39.0 ± 2.3	0.240
Waist Hip Ratio	0.84 ± 0.05	0.93 ± 0.07	<0.001	0.89 ± 0.05	0.92 ± 0.05	0.083
Systolic blood pressure (mmHg)	123.0 ± 16.1	126.0 ± 15.5	0.413	128.7 ± 18.8	135.7 ± 20.1	0.291
Diastolic blood pressure (mmHg)	79.1 ± 10.5	80.3 ± 10.5	0.313	81.8 ± 13.9	87.6 ± 16.1	0.060
Epworth sleepiness scale	7.4 ± 4.4	11.0 ± 4.1	0.006	7.4 ± 4.6	10.6 ± 4.6	0.010
Apnea-hypopnea index (events/h)	1.4 ± 1.6	43.7 ± 24.1	<0.001	1.4 ± 1.5	32.8 ± 22.5	<0.001
RDI (events/h)	7.9 ± 5.9	54.3 ± 22.1	<0.001	8.6 ± 6.8	32.8 ± 22.5	<0.001
Mean SaO_2_ (%)	96.7 ± 0.8	94.7 ± 1.6	<0.001	96.6 ± 1.1	94.3 ± 2.1	<0.001
Minimal SaO_2_ (%)	92.7 ± 2.5	80.0 ± 9.0	<0.001	92.3 ± 2.5	79.3 ± 8.5	<0.001
SaO_2_ < 90% (% Total Sleep Time)	0.01 ± 0.03	6.32 ± 9.2	<0.001	0.01 ± 0.03	10.7 ± 16.8	<0.001
Oxygen desaturation index (events/hr)	4.2 ± 12.8	41.4 ± 25.1	<0.001	3.0 ± 3.0	37.3 ± 22.6	<0.001

* All patients and controls are male; plus-minus values are means ± SD. † The body-mass index is the weight in kilograms divided by the square of the height in meters.

**Table 2:** Baseline characteristics of patients with the obstructive sleep apnea syndrome and controls *.

	Validation Cohort (*n* = 120)				
Characteristic	Control	Mild OSA	Moderate OSA	Severe OSA	*p* Value **
*n*	18	28	27	47	-
M:F (*n*)	14:4	21:7	20:7	42:5	-
Age (yr)	35.8 ± 13.9	40.6 ± 12.6	45.5 ± 15.3	44.7 ± 14.1	0.080
BMI (kg/m^2^) †	22.6 ± 3.6	24.9 ± 2.8	24.9 ± 3.2	27.1 ± 3.8	<0.001
Hypertension (*n*)	4	4	5	21	-
Diabetes (*n*)	1	0	1	3	-
Smoking (*n*)	6	2	6	16	-
Systolic blood pressure (mmHg)	133.4 ± 16.2	128.1 ± 12.8	129.4 ± 14.9	138.1 ± 21.6	0.092
Diastolic blood pressure (mmHg)	81.1 ± 11.8	78.0 ± 8.6	79.3 ± 8.6	85.3 ± 14.7	0.060
Apnea-hypopnea index (events/h)	2.3 ± 1.4	9.8 ± 3.2	22.2 ± 4.5	52.6 ± 20.2	<0.001
Mean SaO_2_ (%)	97.0 ± 1.0	96.1 ± 0.8	96.0 ± 1.1	94.2 ± 2.7	<0.001
Minimal SaO_2_ (%)	90.9 ± 3.6	88.6 ± 3.7	85.7 ± 4.1	78.4 ± 8.3	<0.001
5-HETE (ng/mg creatinine)	25.7 ± 9.4	28.4 ± 8.6	42.2 ± 16.6	45.9 ± 21.2	<0.001
5-oxoETE (ng/mg creatinine)	23.8 ± 7.6	23.8 ± 6.8	36.3 ± 14.7	38.6 ± 18.2	<0.001

* Plus-minus values are means ± SD. ** ANOVA used. † The body-mass index is the weight in kilograms divided by the square of the height.

**Table 3:** Odds ratios of OSA according to the cutoff values of 5-HETE and 5-oxoETE.

	Estimated Odds Ratio (95% CI)				
	No. of Subjects	Unadjusted	*p* Value	Multivariate ^a^	*p* Value
5-HETE (<27.5 ng/mg)	39	reference		reference	
5-HETE (≥27.5 ng/mg)	81	7.60 (2.47–23.37)	<0.001	11.71 (2.55–48.58)	0.001
5-oxoETE (<24.0 ng/mg)	44	reference		reference	
5-oxoETE (≥24.0 ng/mg)	76	3.29 (1.17–9.25)	0.024	4.71 (1.29–17.24)	0.019

Abbreviations: 5-HETE, 5-hydroxyeicosatetraenoic acid; 5-oxoETE, 5-oxo-eicosatetraenoic acid; AHI, apnea-hypopnea index; CI, confidence interval. ^a^ Adjusted for age, gender, body mass index, smoking status, and presence of hypertension and diabetes mellitus.

## Data Availability

The data are contained within the article or the Supplementary Materials.

## Supplementary Materials

The following are available online at https://www.mdpi.com/article/10.3390/antiox10081242/s1.

## Abbreviations

5-HETE, 5-hydroperoxyeicosatetraenoic acid; 5-oxoETE, 5-oxo-6*E*,8*Z*,11*Z*,14*Z*-eicosatetraenoic acid; AHI, apnea-hypopnea index; CPAP, continuous positive airway pressure; HIF-1α, hypoxia-inducible factor 1α; hPBMC, human peripheral blood mononuclear cell; IH, intermittent hypoxia; OSA, obstructive sleep apnea.

## References

[1] Somers V.K., Dyken M.E., Clary M.P., Abboud F.M. (1995). Sympathetic neural mechanisms in obstructive sleep apnea. J. Clin. Investig..

[2] Fichter J., Bauer D., Arampatzis S., Fries R., Heisel A., Sybrecht G.W. (2002). Sleep-related breathing disorders are associated with ventricular arrhythmias in patients with an implantable cardioverter-defibrillator. Chest.

[3] Fein A.S., Shvilkin A., Shah D., Haffajee C.I., Das S., Kumar K., Kramer D.B., Zimetbaum P.J., Buxton A.E., Josephson M.E. (2013). Treatment of obstructive sleep apnea reduces the risk of atrial fibrillation recurrence after catheter ablation. J. Am. Coll. Cardiol..

[4] Kohler M., Stradling J.R. (2010). Mechanisms of vascular damage in obstructive sleep apnea. Nat. Rev. Cardiol..

[5] Young T., Peppard P.E., Gottlieb D.J. (2002). Epidemiology of obstructive sleep apnea: A population health perspective. Am. J. Respir. Crit. Care Med..

[6] Masa J.F., Corral J., Sanchez de Cos J., Duran-Cantolla J., Cabello M., Hernandez-Blasco L., Monasterio C., Alonso A., Chiner E., Aizpuru F. (2013). Effectiveness of three sleep apnea management alternatives. Sleep.

[7] Yoon D.W., Shin H.W. (2020). Sleep tests in the non-contact era of the COVID-19 pandemic: Home sleep tests versus in-laboratory polysomnography. Clin. Exp. Otorhinolaryngol..

[8] Stanke-Labesque F., Back M., Lefebvre B., Tamisier R., Baguet J.P., Arnol N., Levy P., Pepin J.L. (2009). Increased urinary leukotriene E4 excretion in obstructive sleep apnea: Effects of obesity and hypoxia. J. Allergy Clin. Immunol..

[9] Villa M.P., Supino M.C., Fedeli S., Rabasco J., Vitelli O., Del Pozzo M., Gentile G., Lionetto L., Barreto M., Simmaco M. (2014). Urinary concentration of 8-isoprostane as marker of severity of pediatric OSAS. Sleep Breath.

[10] Gozal D., Jortani S., Snow A.B., Kheirandish-Gozal L., Bhattacharjee R., Kim J., Capdevila O.S. (2009). Two-dimensional differential in-gel electrophoresis proteomic approaches reveal urine candidate biomarkers in pediatric obstructive sleep apnea. Am. J. Respir. Crit. Care Med..

[11] Montesi S.B., Bajwa E.K., Malhotra A. (2012). Biomarkers of sleep apnea. Chest.

[12] Conte L., Greco M., Toraldo D.M., Arigliani M., Maffia M., de Benedetto M. (2020). A review of the “OMICS” for management of patients with obstructive sleep apnoea. Acta Otorhinolaryngol. Ital..

[13] Lee H.-S., Kim S.-M., Jang J.-H., Park H.-D., Lee S.-Y. (2021). Serum 5-hydroxyindoleacetic acid and ratio of 5-hydroxyindoleacetic acid to serotonin as metabolomics indicators for acute oxidative stress and inflammation in vancomycin-associated acute kidney injury. Antioxidants.

[14] Lopez-Yerena A., Dominguez-Lopez I., Vallverdu-Queralt A., Perez M., Jauregui O., Escribano-Ferrer E., Lamuela-Raventos R.M. (2021). Metabolomics technologies for the identification and quantification of dietary phenolic compound metabolites: An overview. Antioxidants.

[15] Sabatine M.S., Liu E., Morrow D.A., Heller E., McCarroll R., Wiegand R., Berriz G.F., Roth F.P., Gerszten R.E. (2005). Metabolomic identification of novel biomarkers of myocardial ischemia. Circulation.

[16] Weckwerth W. (2010). Metabolomics: An integral technique in systems biology. Bioanalysis.

[17] Marin J.M., Carrizo S.J., Vicente E., Agusti A.G.N. (2005). Long-term cardiovascular outcomes in men with obstructive sleep apnoea-hypopnoea with or without treatment with continuous positive airway pressure: An observational study. Lancet.

[18] Yoon D.W., Kim Y.S., Hwang S., Khalmuratova R., Lee M., Kim J.H., Lee G.Y., Koh S.J., Park J.W., Shin H.W. (2019). Intermittent hypoxia promotes carcinogenesis in azoxymethane and dextran sodium sulfate-induced colon cancer model. Mol. Carcinog..

[19] Yoon D.W., So D., Min S., Kim J., Lee M., Khalmuratova R., Cho C.H., Park J.W., Shin H.W. (2017). Accelerated tumor growth under intermittent hypoxia is associated with hypoxia-inducible factor-1-dependent adaptive responses to hypoxia. Oncotarget.

[20] Chun Y.S., Choi E., Kim G.T., Lee M.J., Lee M.J., Lee S.E., Kim M.S., Park J.W. (2000). Zinc induces the accumulation of hypoxia-inducible factor (HIF)-1alpha, but inhibits the nuclear translocation of HIF-1beta, causing HIF-1 inactivation. Biochem. Biophys. Res. Commun..

[21] Werz O. (2002). 5-lipoxygenase: Cellular biology and molecular pharmacology. Curr. Drug Targets Inflamm. Allergy.

[22] Demasi M., Cleland L.G., Cook-Johnson R.J., Caughey G.E., James M.J. (2003). Effects of hypoxia on monocyte inflammatory mediator production: Dissociation between changes in cyclooxygenase-2 expression and eicosanoid synthesis. J. Biol. Chem..

[23] Yuan G., Nanduri J., Khan S., Semenza G.L., Prabhakar N.R. (2008). Induction of HIF-1alpha expression by intermittent hypoxia: Involvement of NADPH oxidase, Ca^2+^ signaling, prolyl hydroxylases, and mTOR. J. Cell. Physiol..

[24] Garvey J.F., Taylor C.T., McNicholas W.T. (2009). Cardiovascular disease in obstructive sleep apnoea syndrome: The role of intermittent hypoxia and inflammation. Eur. Respir. J..

[25] Yaggi H.K., Concato J., Kernan W.N., Lichtman J.H., Brass L.M., Mohsenin V. (2005). Obstructive sleep apnea as a risk factor for stroke and death. N. Engl. J. Med..

[26] Kent B.D., Ryan S., McNicholas W.T. (2011). Obstructive sleep apnea and inflammation: Relationship to cardiovascular co-morbidity. Respir. Physiol. Neurobiol..

[27] Grant G.E., Gravel S., Guay J., Patel P., Mazer B.D., Rokach J., Powell W.S. (2011). 5-Oxo-ETE is a major oxidative stress-induced arachidonate metabolite in B lymphocytes. Free Radic. Biol. Med..

[28] Huang Y., Zhu M., Li Z., Sa R., Chu Q., Zhang Q., Zhang H., Tang W., Zhang M., Yin H. (2014). Mass spectrometry-based metabolomic profiling identifies alterations in salivary redox status and fatty acid metabolism in response to inflammation and oxidative stress in periodontal disease. Free Radic. Biol. Med..

[29] Rousseau A.S., Richer C., Richard M.J., Favier A., Margaritis I. (2006). Plasma glutathione peroxidase activity as a potential indicator of hypoxic stress in breath-hold diving. Aviat. Space Environ. Med..

[30] Bierl C., Voetsch B., Jin R.C., Handy D.E., Loscalzo J. (2004). Determinants of human plasma glutathione peroxidase (GPx-3) expression. J. Biol. Chem..

[31] Peters-Golden M., Henderson W.R. (2007). Leukotrienes. N. Engl. J. Med..

[32] Mehrabian M., Allayee H. (2003). 5-lipoxygenase and atherosclerosis. Curr. Opin. Lipidol..

[33] Stanke-Labesque F., Pepin J.L., de Jouvencel T., Arnaud C., Baguet J.P., Petri M.H., Tamisier R., Jourdil J.F., Levy P., Back M. (2012). Leukotriene B4 pathway activation and atherosclerosis in obstructive sleep apnea. J. Lipid Res..

[34] Carpagnano G.E., Kharitonov S.A., Resta O., Foschino-Barbaro M.P., Gramiccioni E., Barnes P.J. (2002). Increased 8-isoprostane and interleukin-6 in breath condensate of obstructive sleep apnea patients. Chest.

[35] Carpagnano G.E., Kharitonov S.A., Resta O., Foschino-Barbaro M.P., Gramiccioni E., Barnes P.J. (2003). 8-Isoprostane, a marker of oxidative stress, is increased in exhaled breath condensate of patients with obstructive sleep apnea after night and is reduced by continuous positive airway pressure therapy. Chest.

[36] Li Y.X., Chongsuvivatwong V., Geater A., Liu A. (2009). Exhaled breath condensate cytokine level as a diagnostic tool for obstructive sleep apnea syndrome. Sleep Med..

[37] Janssen L.J. (2001). Isoprostanes: An overview and putative roles in pulmonary pathophysiology. Am. J. Physiol. Lung Cell. Mol. Physiol..

[38] Bittleman D.B., Casale T.B. (1995). 5-Hydroxyeicosatetraenoic acid (HETE)-induced neutrophil transcellular migration is dependent upon enantiomeric structure. Am. J. Respir. Cell. Mol. Biol..

[39] Gordon E.E., Gordon J.A., Spector A.A. (1991). HETEs and coronary artery endothelial cells: Metabolic and functional interactions. Am. J. Physiol..

[40] Burhop K.E., Selig W.M., Malik A.B. (1988). Monohydroxyeicosatetraenoic acids (5-HETE and 15-HETE) induce pulmonary vasoconstriction and edema. Circ. Res..

[41] Mallat Z., Nakamura T., Ohan J., Leseche G., Tedgui A., Maclouf J., Murphy R.C. (1999). The relationship of hydroxyeicosatetraenoic acids and F2-isoprostanes to plaque instability in human carotid atherosclerosis. J. Clin. Investig..

[42] Strassburg K., Huijbrechts A.M., Kortekaas K.A., Lindeman J.H., Pedersen T.L., Dane A., Berger R., Brenkman A., Hankemeier T., van Duynhoven J. (2012). Quantitative profiling of oxylipins through comprehensive LC-MS/MS analysis: Application in cardiac surgery. Anal. Bioanal. Chem..

[43] Zu L., Guo G., Zhou B., Gao W. (2016). Relationship between metabolites of arachidonic acid and prognosis in patients with acute coronary syndrome. Thromb. Res..

[44] Xu Y.J., Ho W.E., Xu F., Wen T., Ong C.N. (2013). Exploratory investigation reveals parallel alteration of plasma fatty acids and eicosanoids in coronary artery disease patients. Prostaglandins Other Lipid Mediat..

[45] Grant G.E., Rokach J., Powell W.S. (2009). 5-Oxo-ETE and the OXE receptor. Prostaglandins Other Lipid Mediat..

