# Supplementary material for: Urine 5-Eicosatetraenoic Acids as Diagnostic Markers for Obstructive Sleep Apnea
Source: Antioxidants (Basel). 2021 Aug 3;10(8):1242. doi: 10.3390/antiox10081242 (PMC8389315; doi:10.3390/antiox10081242)
Supplement: Supplementary file 1 [file antioxidants-10-01242-s001.zip › antioxidants-1280132.sup.pdf]

Table S1. Characteristics Before and After Treatment With CPAP in 11 Patients with the Obstructive Sleep Apnea Syndrome and Controls.*

	Treatment cohort		
Characteristic	Pre-CPAP	Post-CPAP	*P* value‡
**Male (%)**	100	100	
**Age (yr)**	41.9 ± 6.7	44.8 ± 6.6	0.001
**BMI (kg/m^2)†**	26.5 ± 2.7	26.3 ± 2.7	0.210
**Neck circumference (cm)**	38.9 ± 3.3	39.0 ± 2.2	0.572
**Waist Hip Ratio**	0.93 ± 0.04	0.92 ± 0.04	0.397
**Systolic blood pressure (mmHg)**	137.5 ± 25.7	135.1 ± 17.4	0.449
**Diastolic blood pressure (mmHg)**	93.3 ± 17.2	88.9 ± 9.7	0.423
**Epworth Sleepiness Scale**	12.4 ± 5.2	10.4 ± 5.5	0.138
**Apnea-hypopnea index (events/h)**	42.7 ± 23.1	0.7 ± 1.0	0.003
**RDI (events/h)**	50.9 ± 19.4	2.4 ± 2.6	0.003
**Mean SaO_2 (%)**	93.5 ± 2.7	97.2 ± 0.6	0.003
**Minimal SaO_2 (%)**	75.1 ± 7.5	93.8 ± 1.0	0.003
**SaO_2 < 90% (% Total Sleep Time)**	16.9 ± 16.8	0.0 ± 0.0	0.003
**Oxygen desaturation index (events/hr)**	44.8 ± 23.1	0.8 ± 0.9	0.003
**5-HETE (mass abundance)**	14461 ± 6484	9147 ± 2976	0.010
**5-oxoETE (mass abundance)**	26635 ± 13122	15976 ± 7500	0.026

* All patients and controls are male; Plus-minus values are means ± SD. † The body-mass index is the weight in kilograms devided by the square of the height in meters. ‡ *P* values were calculated by paired Wilcoxon signed rank test.

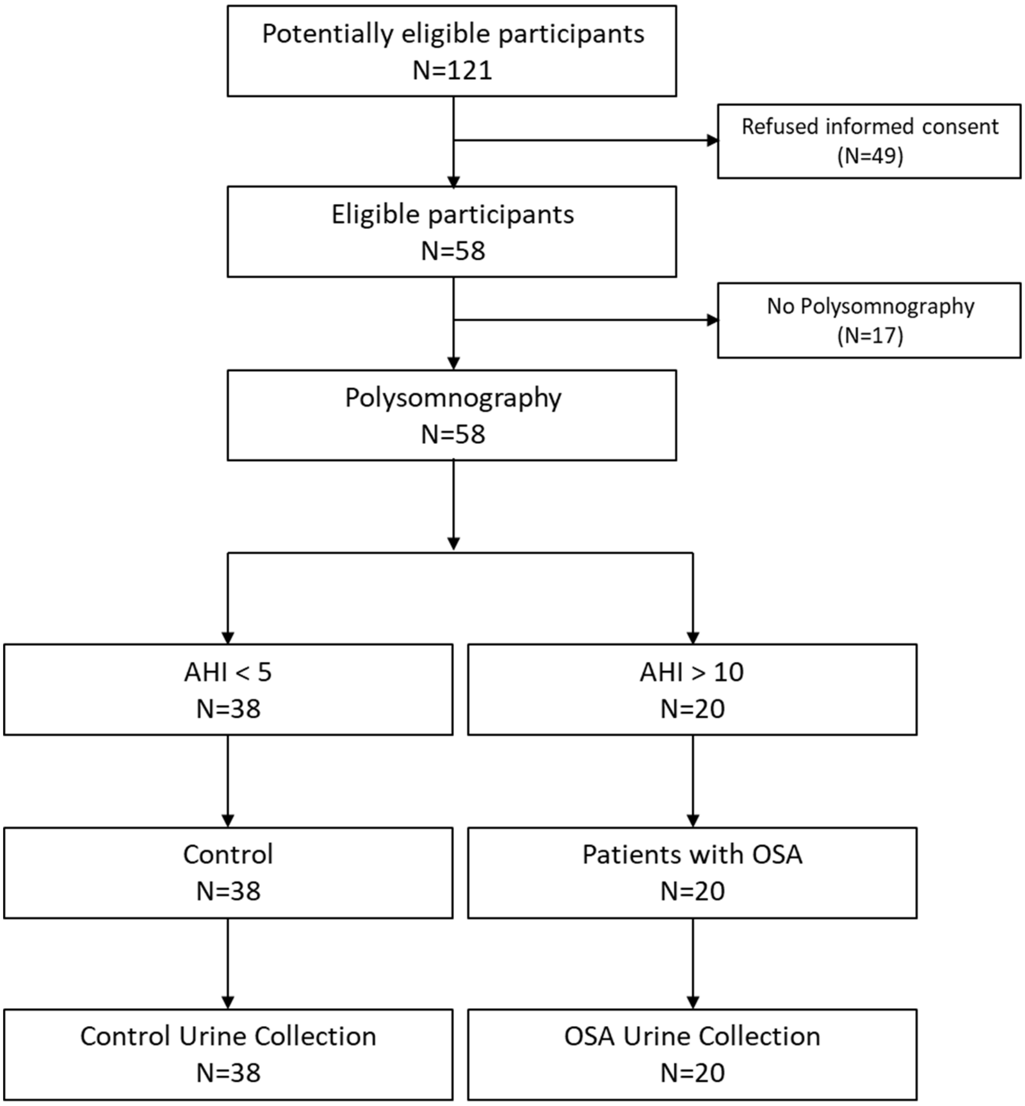

Figure S1: A flow chart of pilot cohort.

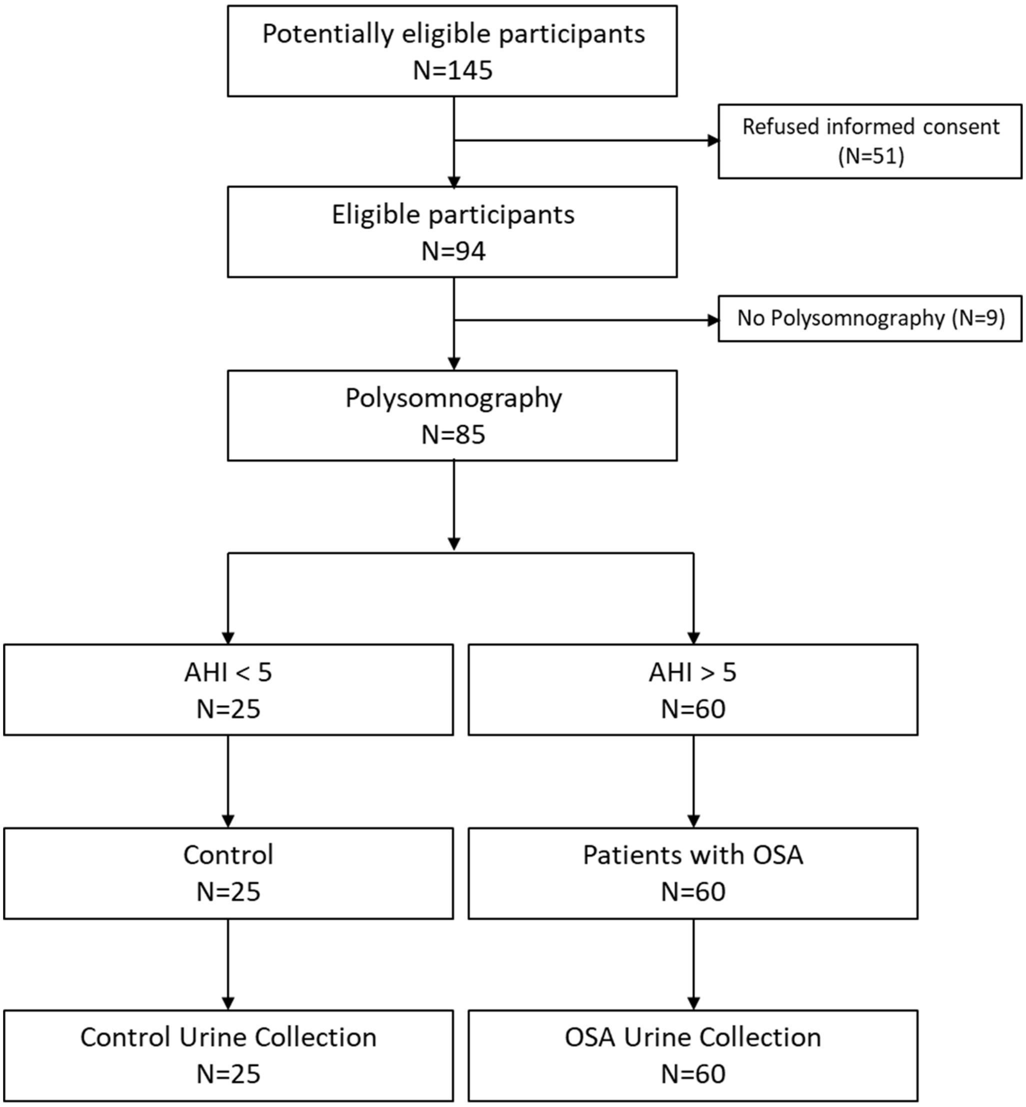

Figure S2: A flow chart of verification cohort.

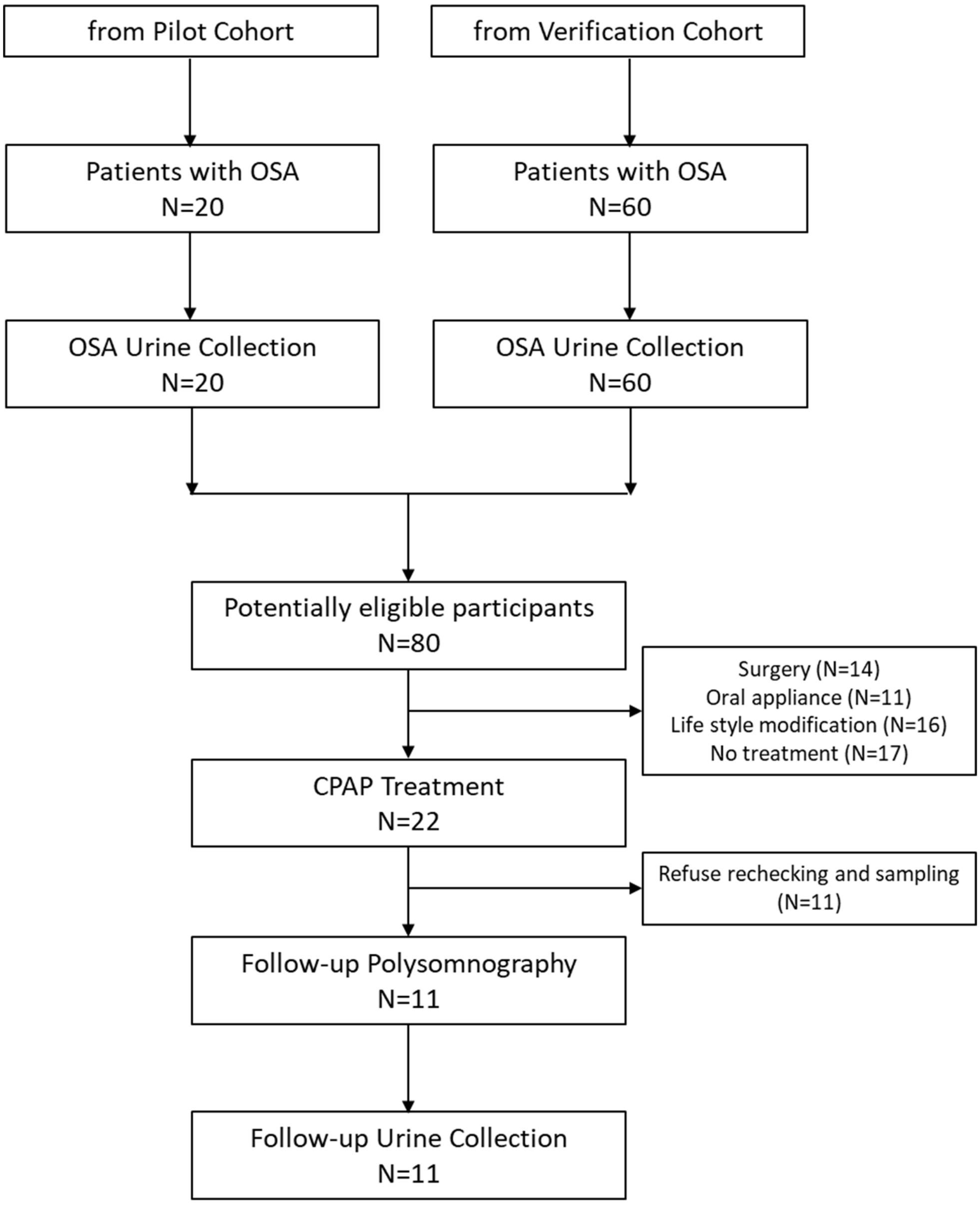

Figure S3: A flow chart of post-CPAP.

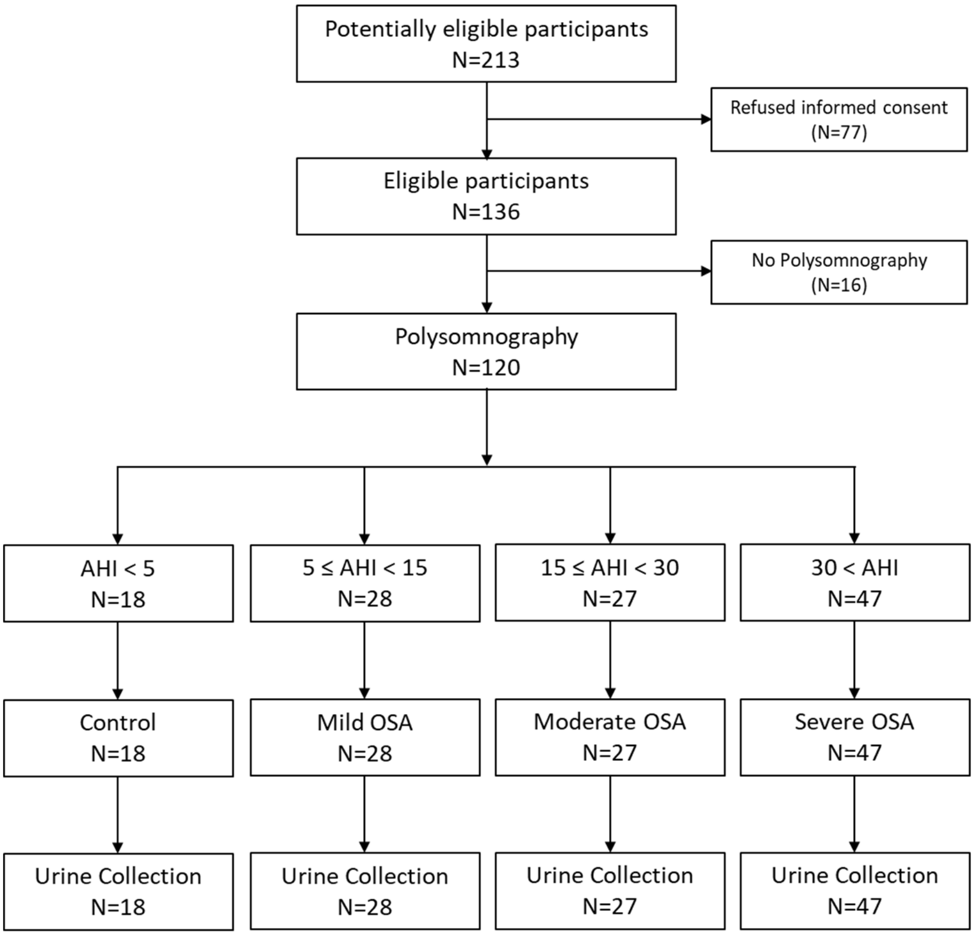

Figure S4: A flow chart of validation cohort.

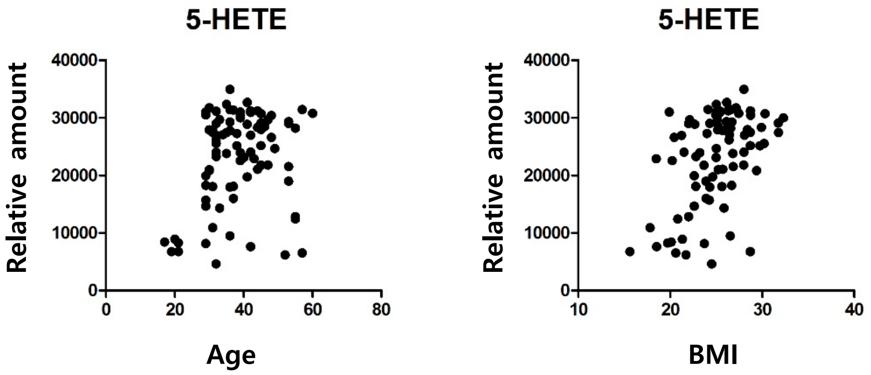

(a)

	Pearson correlation		Partial correlation		
	R	*P*	Control variables	R	*P*
**5-HETE and AHI**	0.571	<0.001	Age, BMI	0.441	<0.001
**5-HETE and lowest saturation**	-0.625	<0.001	Age, BMI	-0.502	<0.001

(b)

Figure S5. (**a**) Correlation between 5-HETE levels and Age (*left*) or body mass index (BMI) (*right*) in the verification cohort. (**b**) Pearson's or partial correlation studies between 5-HETE abundance and apnea-hyponea index (AHI) or the lowest oxygen saturation. BMI means the body mass index.

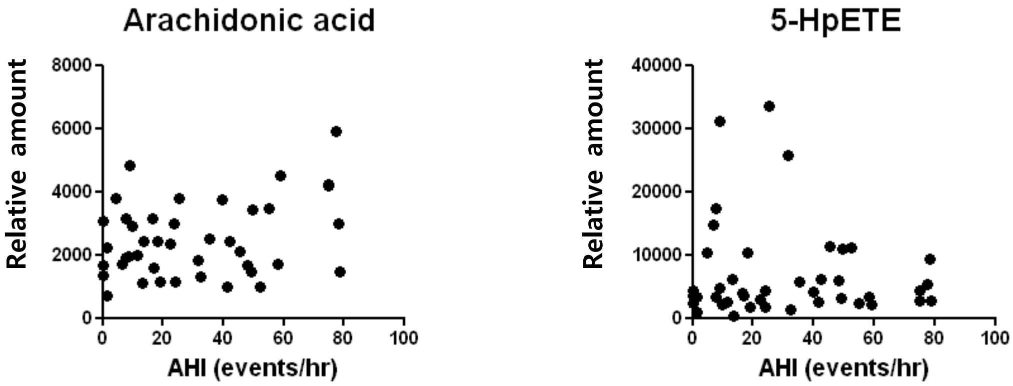

Figure S6. Correlation between arachidonic acid (*left*) or 5-HpETE (*right*) and AHI from polysomnographic studies in the pathway cohort.

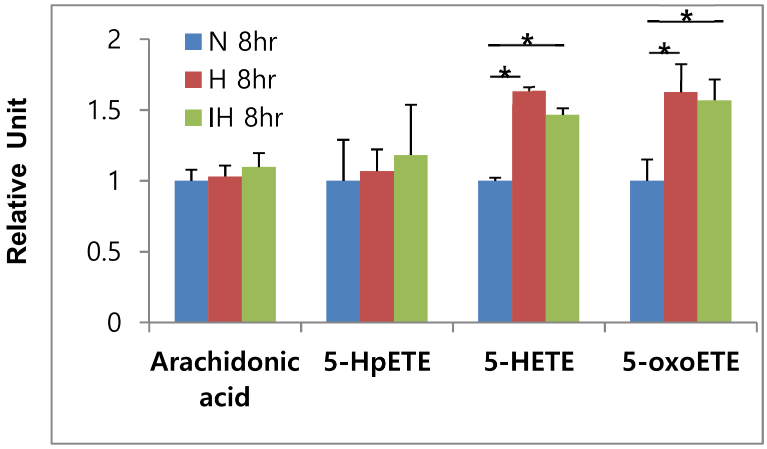

(**a**)

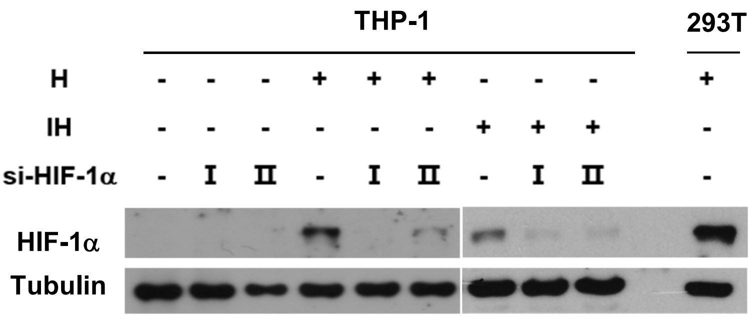

(**b**)

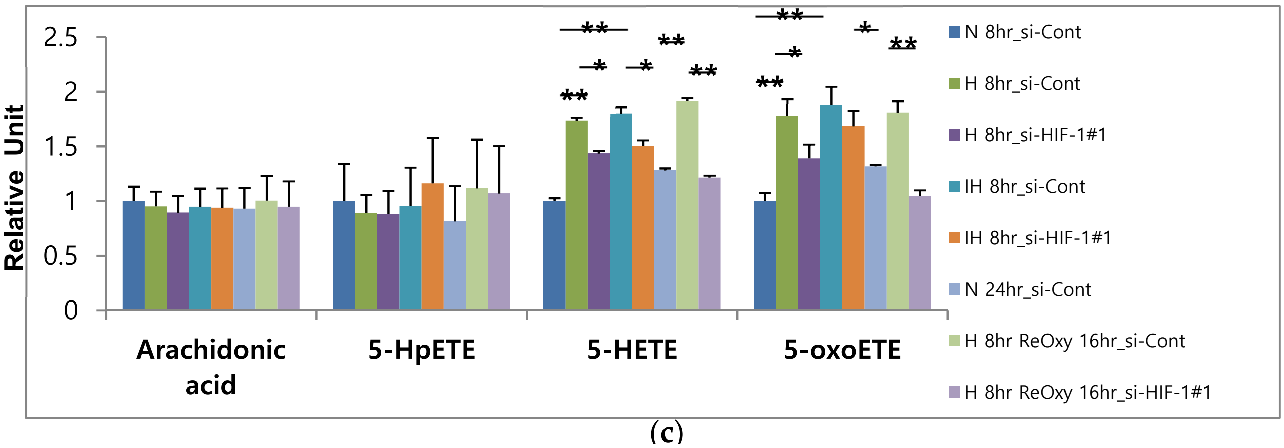

(c)

Figure S7. Augmented Secretion of 5-HETE and 5-oxo-ETE from Human Monocytes by Hypoxia. (**a**) Levels of arachidonic acid and its derivatives secreted from human primary mononuclear cells by 8 hours of normoxia (21% O_2), continuous (1% O_2) or intermittent hypoxia. For intermittent hypoxia, cells were exposed to 8 cycles of hypoxia and normoxia, and each cycle consisted of 10 minute-hypoxia followed by 50 minute-reoxygenation. (**b**) Results of Western blotting of the expression of HIF-1a in THP-1 or HEK293T cells by 8 hours of normoxia, continuous or intermittent hypoxia. (**c**) Silencing of HIF-1a reduced the secretion of 5-HETE and 5-oxo-ETE from human primary mononuclear cells by hypoxia (1% O_2 for 8 hr) or reoxygenation (21% O_2 for 16hr) after hypoxia (1% O_2 for 8hr).

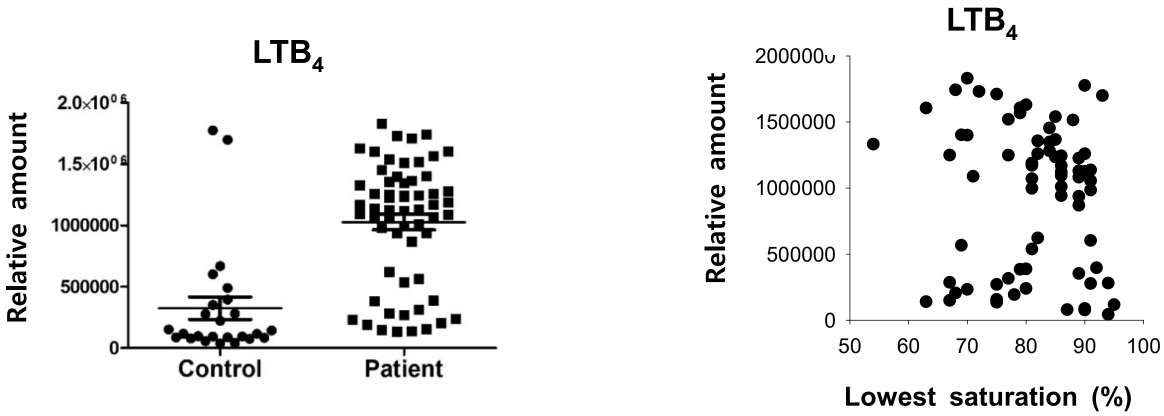

Figure S8. Level of LTB_4 in a different group as a verification cohort – 25 healthy controls, 60 OSA patients. The horizontal lines indicate means, and I bars standard deviations (*left*). Correlation between the level of LTB_4 and the lowest oxygen saturation from polysomnographic studies in the verification cohort (*right*).
